# The Effects of an Appropriate Behavior Program on Elementary School Children Social Skills Development in Physical Education

**DOI:** 10.3389/fpsyg.2019.01998

**Published:** 2019-09-20

**Authors:** Pedro Gil-Madrona, Eva Cristina Gutiérrez-Marín, Marcos Cupani, Amaury Samalot-Rivera, Arturo Díaz-Suárez, Guillermo F. López-Sánchez

**Affiliations:** ^1^Faculty of Education of Albacete, University of Castilla-La Mancha, Albacete, Spain; ^2^Universidad Nacional de Córdoba, Córdoba, Argentina; ^3^The College at Brockport, The State University of New York, New York, NY, United States; ^4^Faculty of Sport Sciences, University of Murcia, Murcia, Spain

**Keywords:** appropriate behaviors, social skills, games, sports, physical education, elementary education, fair play

## Abstract

The purpose of this study was to see the effects of an appropriate behavior development program in relation to the improvement of fair play and social skills behaviors of elementary school children during the physical education class. The participants of this study were 204 students from 5th and 6th grade from seven different public schools in Castilla–La Mancha, Spain. Data was collected through a questionnaire to measure students’ pre and post appropriate behaviors when winning, appropriate behaviors when loosing, appropriate behaviors during the game, fair play skills and social skills, during the physical education class. Results from this study revealed that participants improved their behaviors with the implementation of the intervention (appropriate behavior program), generating positive changes in students’ attitudes and social skills during physical education class. In relation to children gender, girls had better scores than boys in relation to appropriate behaviors when losing. Further, when comparing students’ age, those from 10 to 11 years old scored higher in fair play behaviors and social skills. In conclusion, the intervention program was effective to improve the behaviors of the school children, generating positive changes in their attitudes and social skills during physical education.

## Introduction

Focusing on the development of values and appropriate social skill during physical education and sports is extremely important (Samalot-Rivera, 2007; Samalot-Rivera and Porretta, 2012). Physical education is considered one of the school topics with the potential to develop these behaviors (Moore et al., 1995; Vidoni, 2003; Ponce, 2006; Samalot-Rivera and Porretta, 2009, 2012). For years now, physical education and sports had been considered appropriate context to develop a healthy lifestyle, including the development of appropriate behaviors, values and social skills (Hellison, 2003). Further, physical education is seen as one of the schools’ subject with greater impact of mental, emotional and physical development of students (Ponce, 2006). Weinberg (2011) mentions that the participation in sports, facilitate numerous interpersonal relation situations and the development of interpersonal and social skills.

Learning to co-exist should be one of education main objectives. We must promote values like tolerance, solidarity, and mutual respect though the physical education class because is an appropriate and ideal environment to achieve this (Eldar et al., 2006; Gil-Madrona et al., 2006). The physical education curriculum offers specific opportunities, that are not possible in other subjects (Siedentop, 1991; Laker, 2000; Siedentop et al., 2004).

Bloom and Smith (1996) and Hellison (2003) mentioned that neither teachers or coaches can assume that children will learn values and social skills by just participating in the activities without the proper planning and implementation during physical education and sports. Further, Velázquez-Callado (2004) consider that teachers who choose to teach using cooperative activities help students learn the objective of the activities and developing simultaneously social skills, auto control skills and empathy toward their peers in the physical education class.

During the last years, there had been an increasing interest to examine the effect of physical activity and sport programs on students’ social development experiences during the physical education class. Cooperative learning had been one of the programs used by Fernández Río (2003), in wish he demonstrated improvements on student’s social abilities like trust, enjoyment, and friendship using cooperate learning activities. Cecchini et al. (2008a) found out that physical activity offers a unique opportunity for peer interaction, respect and tolerance. For this reason, all personnel working with students or athletes, should have the proper training to implement and teach these values. This had inspired many researchers during the last decade to develop and design intervention programs to promote the moral development of students and athletes during physical education class and sport related activities (Escartí et al., 2005, 2006; Ruiz et al., 2006).

Gil-Madrona et al. (2016), examined the effectiveness of a physical education program focused on the teaching of social skills and values, fair play, social relationships, effort, self-discipline, and self-control in the social affective context on students from 6th to 8th grade. They concluded that when social abilities and values interventions are implemented, students improve their knowledge about how to positively behave in their physical education class and physical activity context. Further, Danish and Nellen (1997) who presented a model for the development of skills for life, with the objective of using physical activity and sport for the development of personal and social competencies in children and youth, defend that psychosocial acquired abilities can be transferred to other domains as long as the experiences are designed and focused with same purpose (Madrid-López et al., 2016). However, the transfer of the acquired qualities in sport to other areas of life is a controversial topic and has not been proven yet (McPherson, 1986; Heinemann, 1992; García Ferrando et al., 2017).

Some studies had focused on comparing the attitudes of males and females on sport. For example, Derry (2002) affirms that literature demonstrate boys caring more about playing and winning, and girls are more interested in learning the proper ways to play, what implies having better skill and tactic development, and social interactions. We must take this evidence in consideration when planning social skill instruction and change behaviors during the physical education class.

The purpose of this study was to know the effects of a program named Delfos (Cecchini et al., 2008a, b), in relation to the improvement of appropriate behaviors during and after physical activity or sport, fair play and social skills. The program Delfos is a pedagogical intervention program designed to develop appropriate behaviors when winning, appropriate behaviors when loosing, appropriate behaviors during the game, fair play skills and social skills, in youth during physical education class. To our knowledge, this is the first study analyzing these aspects in Castilla–La Mancha (Spain).

## Materials and Methods

### Participants

Sample was composed of 204 students from 5th (21.7%) and 6th (78.9%) grade, between 10 and 13 years old (*X* = 11.22; *SD* = 0.70) with 46.1% girls and 53.9% boys. Students were from seven different public schools in Albacete Spain. Sample selection was done by convenience. All public schools from urban and disadvantage areas from Albacete Spain were taken in consideration. All subjects gave assent and their parents provided their informed consent for inclusion before they participated in the study. The protocol was approved by the University of Castilla–La Mancha Research Ethics Committee and by the Ethics Committees of the schools participating in the study.

### Data Collection

A validated questionnaire titled Appropriate Behaviors in Physical Education and Sport by Samalot-Rivera and Madrona, was used to collect data in this study. This questionnaire is composed by 32 items with a 5 level Likert scale (1- Never, 2-Sometimes, 3- Occasionally, 4- Most of the Time, and All the Time). The items were grouped from a theoretical point of view and were divided in the following groups: appropriate behaviors when winning, appropriate behaviors when loosing, appropriate behaviors during the game, fair play skills and social skills. Reliability of the instrument was measured by Alpha Cronbach obtaining an adequate 0.883.

### Procedures

An experimental pre and post-test with control groups was used Hernández-Sampieri et al. (2003). The Delfos program was used as the intervention of this study. The Delfos program does have an educative intervention with pedagogical teaching strategies, organization and session designed with the purpose of improving fair play, increase levels of self-control and modify inappropriate behaviors in youth. The principal values associated with Delfos program are student wellbeing, personal development, effort and self-management (Cecchini et al., 2008a, b).

There is no doubt that any program to be implemented, needs to be tested with the purpose of knowing to what extent the program is effective (Hernández-Mendo and Anguera, 2001). For this reason, we decided to test this program to see the effects that have in youth by using a questionnaire about Appropriate Behavior During Physical Education and Sport developed by Samalot Rivera and Pedro Gil Madrona, validated by Gutiérrez-Marín et al. (2017). As part of this program, students needed to complete and sign a behavior contract where they compromise to improve and decrease their aggressive behaviors, eliminate certain conducts of putting other down and increase the capacity to avoid and solve conflicts in sport related activities and in their daily living.

Delfos program structure the physical education classes this way:

(a) Discussion of the social skill: in this phase of the program a discussion about the social skills to be worked was conducted. A discussion of the attitudes and social skills expected as part of the program objectives and values took place.
(b) Activation phase: in this phase of the program the focus was on providing students the opportunity to be physically active.
(c) Confrontation phase: in this phase, students were given constant feedback about their behaviors during class.
(d) Reflection phase: in this phase is when teachers and students analyze possible moral conflicts during the class sport or activity played and come up with solutions.
(e) Transition phase: the objective of this phase was to make sure that students understood the importance of the learned values and social skills will have in their lives. This was discussed during the reflection phase at the end when they were asked to please continue using the discussed and learned values in their communities.

The intervention program was conducted in the physical education class for a total of 20 class sessions of 45 min each. This program was designed with the purpose to help students learn appropriate sport and fair play behaviors before, during and after games.

### Analysis

A 2 × 2 factorial design with pre and post-test design was used for this study. The first factor references student’s gender (male and female). The second factor references the age of the participants (10 to 11 and 12 to 13 years old). Participants were measured pre and post treatment. For data analysis SPSS for Windows version 19.0 was used. With the objective to prepare data for the analysis we examined: (a) the patterns on the lost data to estimate if its respond to a random distribution (Tabachnick and Fidell, 2001), (b) the mean, standard deviation, asymmetry and kurtosis of each one of the variables considered in this study, (c) the presence of univariate and multivariate atypical cases, and (d) the presence of multicollinearity among variables (Kline, 2011). As a criterion to evaluate index of asymmetry and kurtoris it was considered to be excellent values between + 1,00 years −1,00, and as fair values inferior to + 2,00 years −2,00. Later, atypical univariate cases were identified through the standard results of each one of the variables (*z* > 3.29, *p* < 0.001).

A Mahalanobis test with *p* < 0.001 was conducted with the finality of discard multivariate atypical cases (Tabachnick and Fidell, 2001). A last analysis consisted of a multicollinearity test between the variables (Kline, 2011), this to estimate the existence of redundant variables (0.90 correlations or higher).

A variance analysis of repeated measures (ANOVA) was done. Group and independent factors were gender and age (children between 10 and 11 and 12 to 13 years old). Results obtained between pre and post-test were considered repeated measures. Box’s *M* test was used to compare variation in multivariate samples. Partial square eta (η^2^) was used to estimate the size effect and the alfa values was set on 0.05. The effects of the significant interactions given by ANOVA results were explored by a Bonferroni *post hoc*.

## Results

### Data Preparation

The percentage of the lost cases for not responding to some of the items did not overcome the 5%. Because this was such a small percentage, it was decided to impute those by a central tendency measurement (mode) of the completed responses of one of the participants in the same scale. This method had demonstrated on lost cases to promote the conceptual attractive balance of precision and simplicity (Shrive et al., 2006). The selection of impute by was due to the fact that we tried to have the five (discrete) response options of the scale. The asymmetry and kurtosis indices of the variables under study were obtained and varied between −1.25 to −0.22 and between −0.34 to 1.84, respectively, which can be considered as acceptable. Six atypical univariate cases were detected (*z* = ± 3.29), of which four are multivariate atypical cases. The elimination of these atypical cases improves the distribution of the variables (indices of asymmetry between −0.93 to −0.24 and of kurtosis between −0.27 to 0.71). The correlations between the variables varied between *r* = 0.24 to 0.61, which can be concluded that there are no problems of multicollinearity.

### Preliminary Analyses

A *t*-test of mean difference was applied for independent groups in relation to the age and gender of the participants. Regarding the participants gender, it can be observed that there are significant differences in the variables Social Skills (*d* = 0.37). Female students scored higher on these variables than male students. In relation to age, it can be observed that there are significant differences in the variables Appropriate skills when losing (*d* = 0.36), Fair Play Skill (*d* = 0.38) and Social Skill (*d* = 0.31). The group of students between 10 and 11 years old scored higher on these variables than those students between 12 and 13 years old (Table 1).

**TABLE 1:** Mean differences by sex and age group.

	**Sex**							**Age group**						
								
	**Females**		**Males**					**10–11**		**12–13**				
	**(*n* = 93)**		**(*n* = 105)**					**(*n* = 129)**		**(*n* = 69)**				
										
**Variables**	***M***	***SD***	***M***	***SD***	***t***	***p***	***d***	***M***	***SD***	***M***	***SD***	***t***	***p***	***d***
ABL	20.02	3.43	19.18	3.30	1.76	0.08	0.25	20.00	3.34	18.78	3.33	2.45	0.02	0.36
ABW	19.04	3.49	18.57	3.24	0.99	0.33	0.14	18.71	3.42	18.96	3.25	–0.50	0.62	0.07
ABDG	21.42	2.56	20.94	2.63	1.29	0.20	0.18	21.23	2.55	21.04	2.70	0.49	0.63	0.07
FPS	21.69	2.40	21.26	2.80	1.16	0.25	0.16	21.81	2.39	20.81	2.92	2.58	0.01	0.38
SK	53.18	4.45	51.27	5.67	2.62	0.01	0.37	52.73	4.72	51.12	5.92	2.09	0.04	0.31

*M, Mean; SD, Standard Deviation; ABL, Appropriate behaviors when loosing; APW, Appropriate behaviors when winning; ABDG, Appropriate behaviors during game; FPS, Fair play skills; SK, Social skills.*

### ANOVA of Repeated Measures

#### Appropriate Behaviors When Loosing

**FIGURE 1:**
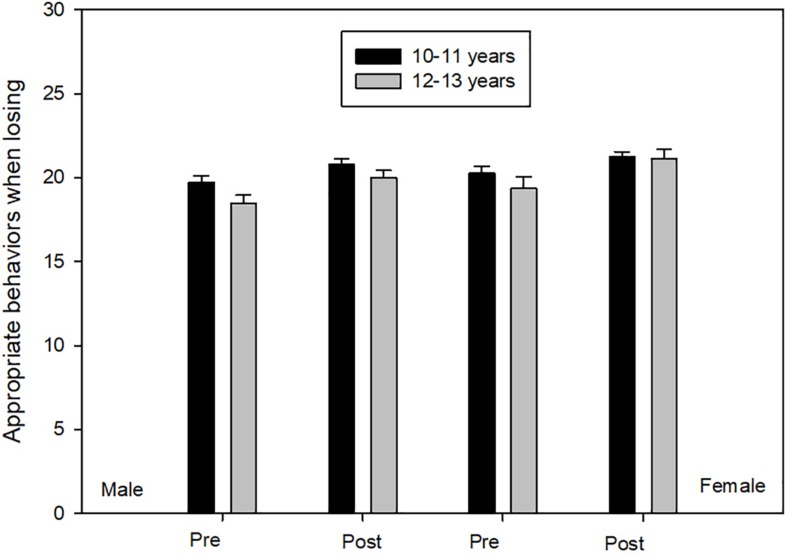
Appropriate behaviors when loosing.

Box’s *M* test was significant (*F* = 3.182, *p* ≤ 0.001), however, Box’s *M* test has been criticized for being overly sensitive for large sample sizes, it can detect even small departures from homogeneity. In other words, it will report a statistically significant result when one does not exist, but they do not increase the probability of type I error (Hahs-Vaughn, 2016). ANOVA measures demonstrated the principal effects of time [*F*(1,194) = 28.689, *p* ≤ 0.01, η^2^ = 0.13, 1-β = 1.00] and gender [*F*(1,194) = 3.83, *p* ≤ 0.05, η^2^ = 0.02, 1-β = 0.50]. Female participants, when compared with male participants, scored significantly higher in appropriate behaviors when losing. In the other hand, post-test scores were significantly higher than pre-test for all four groups (Figure 1).

#### Appropriate Behaviors When Winning

**FIGURE 2:**
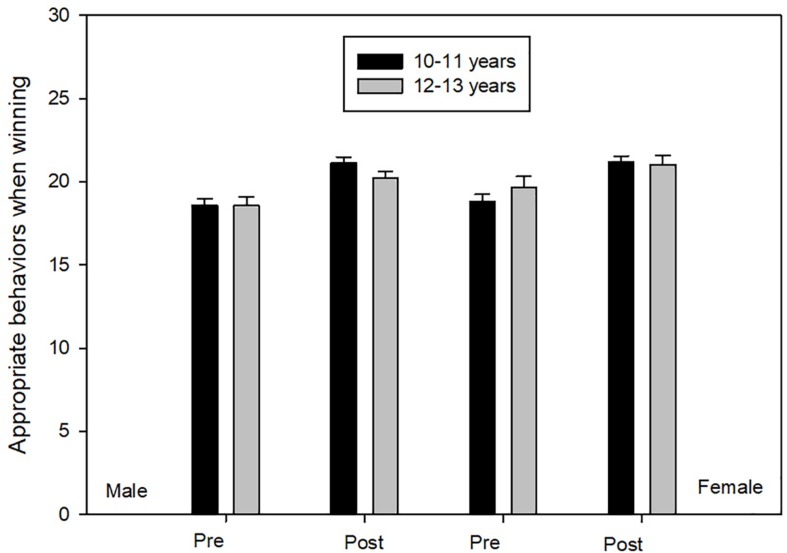
Appropriate behaviors when winning.

Box’s *M* test was no significant (*F* = 0.424, *p* ≥ 0.92). ANOVA scores demonstrated principal effects of time [*F*(1,194) = 49.095, *p* ≤ 0.01, η^2^ = 0.20, 1-β = 1.00]. Participants scored significantly lower on post-test an all four groups in comparison to pre-test (Figure 2).

#### Appropriate Behaviors During Game

**FIGURE 3:**
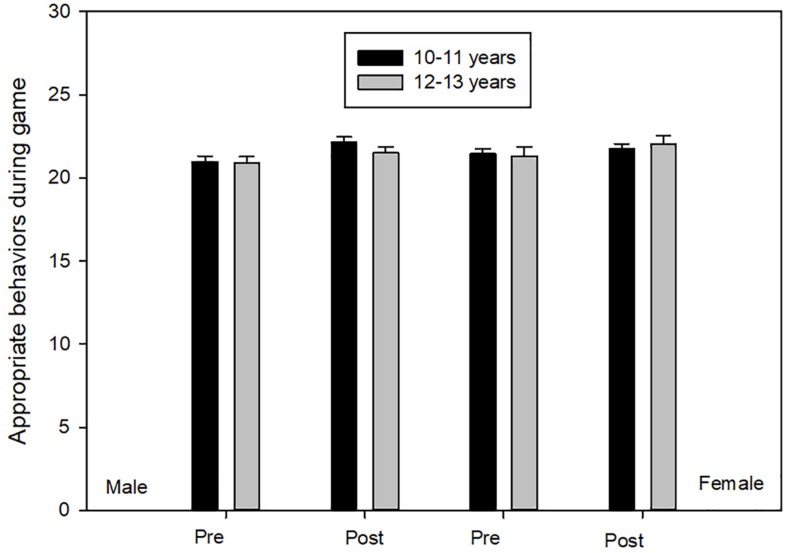
Appropriate behaviors during game.

Box’s *M* test was no significant (*F* = 1.356, *p* ≥ 0.20). ANOVA scores demonstrated principal effects of time [*F*(1,194) = 9.944, *p* ≤ 0.05, η^2^ = 0.05, 1-β = 0.88]. Participants scored significantly higher on post-test on all four groups in comparison to pre-test (Figure 3).

#### Fair Play

**FIGURE 4:**
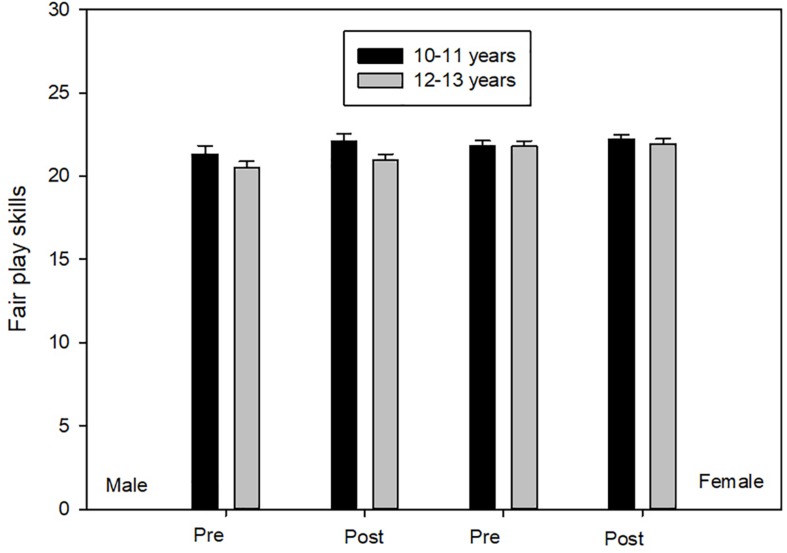
Fair play skills.

Box’s *M* test was no significant (*F* = 1.560, *p* ≥ 0.12). ANOVA scores showed main effects of Time [*F*(1,194) = 3.789, *p* ≤ 0.05, η^2^ = 0.02, 1-β = 0.49] and Age [*F*(1,194) = 5,540, *p* ≤ 0.02, η^2^ = 0.03, 1-β = 0.65]. Participants aged 10 to 11, compared to their peers aged 12 to 13, scored significantly higher on fair play skills. On the other hand, in post test scores, in the four groups, were significantly higher than pre-test scores (Figure 4).

#### Social Skills

**FIGURE 5:**
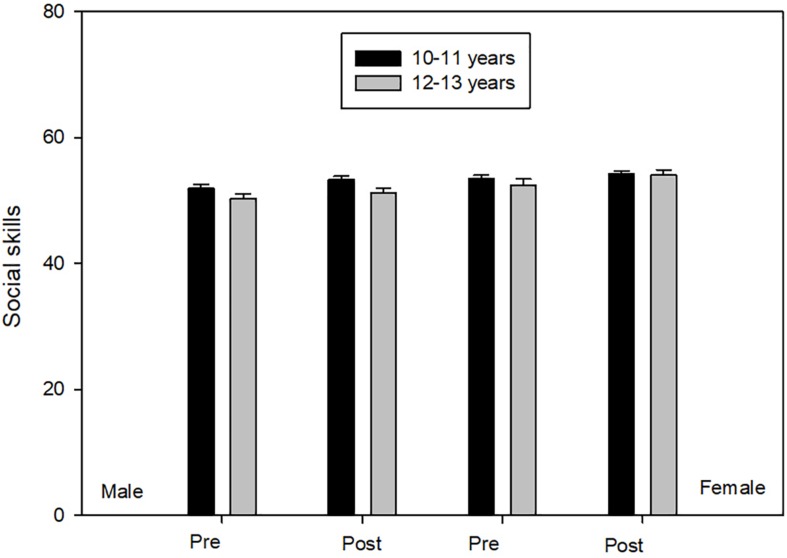
Social skills.

Box’s *M* test was significant (*F* = 2.865, *p* ≤ 0.02). ANOVA scores showed main effects of Time [*F*(1,194) = 8,351, *p* ≤ 0.01, η^2^ = 0.04, 1-β = 0.82], Gender [*F*(1,194) = 8,717, *p* ≤ 0.01, η^2^ = 0.04, 1-β = 0.83] and Age [*F*(1,194) = 3.856, *p* ≤ 0.05, η^2^ = 0.02, 1-β = 0.50]. Participants from 10 to 11 years old, compared to their peers from 12 to 13 years old, scored significantly higher in social skills. However, post test scores, in the four groups, were significantly higher than in the pre-test (Figure 5).

## Discussion

This is a novel study because, to our knowledge, this is the first study in Castilla–La Mancha (Spain) analyzing the impact of the program Delfos on students’ appropriate behaviors when winning, losing and during games, fair play and social skills in the physical education class. Through this study it can be evidenced the positive impact that the implementation of the program Delfos had on the development and transfer of students’ appropriate behaviors when winning, losing and during games, fair play and social skills in the physical education class. Data revealed the improvement of positive attitudes as well as their social skills and fair play versus being carried away with games difficult situations and having negative ones. Nevertheless, it is important to consider that previous studies have indicated that the transfer of the acquired qualities in sport to other areas of life is a controversial topic and has not been proven yet (McPherson, 1986; Heinemann, 1992; García Ferrando et al., 2017).

The results of this study demonstrated that students had significantly better scores after the program was implemented. This results provide evidence that playing a sport is not necessarily what help students improve on their behaviors, but rather the context or intervention during the sport activity is what make the difference, helping students to improve their behaviors, values and social skills (Cecchini et al., 2004, 2005). Cecchini et al. (2005), had similar results when they addressed youth at risk behaviors with the same objectives of this study. It is evident that literature provide enough evidence to believe that this type of program can be used as a preventive measure to address problems related to misconduct and lack of social skills and values.

Changes in attitudes and values (appropriate behaviors) will occur easier when appropriate planning to develop these behaviors in physical activity context are implemented (Samalot-Rivera and Vidoni, 2015). As mentioned many times in the literature, physical education and sports are attractive environments in which these type of interventions can be implemented creating positive behavior changes in students (Vidoni and Ulman, 2012; Samalot-Rivera and Vidoni, 2015). Further, Brustad and Arruza (2002), mentioned that sports events are a good context to develop social skills and values because it offers many opportunities for decision making and personal interaction.

The main strength of the present study is the implementation and evaluation of Delfos program in a new sample of Castilla–La Mancha (Spain), analyzing its impact on students’ appropriate behaviors when winning, losing and during games, fair play, and social skills in the physical education class. This study presents also some limitations. This data does provide good improvement of results from pre and post data after the intervention, but to see the reliability of the program we could have compared to a control group or use a parallel measurement scale to control the differences between pre and post intervention. This study focused on assessing the educational possibilities of sport, but future studies should also assess the educational possibilities of other activities, such as music or art. Finally, new qualitative studies are needed to know if aspects such as class incidents, differences between teachers, or differences between types of schools might affect the implementation of the Delfos program.

## Conclusion

Students are an important part of the teaching learning process and must be part of it by interacting with class content, peers and the established classroom rules. This way students will learn how to deal with classroom conflict resolution. In this study we were able to observe positive changes in students’ target behaviors after the implementation of the program that consisted of 20 sessions of 45 min each. It was the objective of the program to help students improve in their values and social skills and not necessarily in their sport related skills. After analyzing survey data, females had higher scores than males in appropriate behavior when losing and overall social skills. On the other hand, when comparing age groups, those students from 10 to 11 years old scored significantly better on fair play and social skills, in comparison to those from 12 to 13 years old. Therefore, we suggest that these competencies may be transferred to other domains and may be maintained over time by implementing programs like this, and we also suggest to measure students’ behaviors with the Appropriate Behavior Physical Education and Sport questionnaire overtime. This way teachers might reinforce the development of these competencies for students social and personal well-being (Gutiérrez-Marín et al., 2017).

## Data Availability

The datasets generated for this study are available on request to the corresponding authors.

## Ethics Statement

The studies involving human participants were reviewed and approved by the Research Ethics Committee of the Public School Calar Del Mundo. Written informed consent to participate in this study was provided by the participants’ legal guardian/next of kin.

## Author Contributions

All authors listed have made a substantial, direct and intellectual contribution to the work, and approved it for publication.

## Conflict of Interest Statement

The authors declare that the research was conducted in the absence of any commercial or financial relationships that could be construed as a potential conflict of interest.
